# Microbiome-Guided Maternal and Child Nutrition: A Structured Narrative Review of Indigenous African Foods, Fermented Foods, and Emerging Evidence

**DOI:** 10.3390/ijms27198503

**Published:** 2026-09-23

**Authors:** Alice Njolke Mafe, Dietrich Büsselberg

**Affiliations:** 1 Department of Biological Sciences, Faculty of Sciences, Taraba State University, Main Campus, Jalingo 660101, Taraba State, Nigeria; mafealice1991@gmail.com; 2 Department of Physiology and Biophysics, Faculty of Medicine, Qatar Campus, Weill Cornell Medicine Qatar, Education City, Qatar Foundation, Doha Metropolitan Area, Doha P.O. Box 22104, Qatar

**Keywords:** maternal–infant axis, dysbiosis, short-chain fatty acids, nutritional resilience, functional metabolites, immune homeostasis, precision nutrition, early-life programming

## Abstract

The gut microbiota is recognized as important to maternal and child health through associations with immune maturation, metabolic programming, barrier integrity, and early-life disease susceptibility. Indigenous food systems, fermented foods, probiotics, and postbiotics may influence gut microbial communities and functions, suggesting potential avenues for investigation into nutritional resilience, particularly in low-resource settings. This review synthesizes evidence on microbiome-informed maternal and child nutrition, focusing on indigenous foods, fermented foods, probiotics, postbiotics, and microbiome-mediated mechanisms. Literature was searched in PubMed, Scopus, Web of Science, ScienceDirect, and Google Scholar for studies, systematic reviews, and meta-analyses published between 2015 and 2025. Evidence indicates that microbiome-directed nutritional interventions may influence microbial metabolites, particularly short-chain fatty acids, and pathways associated with barrier integrity, immune regulation, and cellular signaling, including adenosine monophosphate-activated protein kinase (AMPK), mechanistic target of rapamycin (mTOR), mitogen-activated protein kinase (MAPK), nuclear factor kappa-light-chain-enhancer of activated B cells (NF-κB), toll-like receptor (TLR), and peroxisome proliferator-activated receptor (PPAR). Artificial intelligence, multi-omics, and precision nutrition may support the investigation and future development of more targeted approaches. However, gaps remain regarding population-specific microbiome profiles, standardized probiotic and postbiotic formulations, longitudinal maternal–infant relationships, and evidence from African and other low-resource settings. The Microbiome-Informed Nutrition Framework (MINF) is proposed as a hypothesis-generating model integrating indigenous food systems, microbiome-directed interventions, precision nutrition, and community-based primary healthcare as potential areas for future research, rather than established clinical or public health interventions.

## 1. Introduction

### 1.1. Maternal and Child Nutrition and the Microbiome

Maternal and child malnutrition remains a major global public health challenge, contributing to maternal, neonatal, and under-five morbidity and mortality [1,2]. The burden includes undernutrition, micronutrient deficiencies, overweight, obesity, and diet-related metabolic disorders, particularly in low- and middle-income countries [3,4]. Conversely, excessive energy intake, particularly when dominated by energy-dense but nutrient-poor foods, may contribute to excessive gestational weight gain, maternal obesity, gestational metabolic disorders, and adverse pregnancy outcomes [5]. Maternal nutrition before conception, during pregnancy, and throughout lactation influences fetal growth and early-life development, while the first 1000 days represent a critical period for growth, immune maturation, and neurodevelopment [6,7]. Increasing evidence indicates that nutritional outcomes are influenced not only by dietary intake but also by interactions among diet, the gut microbiota, host metabolism, and immune function [8]. The gut microbiota contributes to nutrient metabolism, immune development, epithelial integrity, and resistance to pathogens [9,10]. Early microbial colonization is influenced by maternal microbiota, delivery mode, breastfeeding, antibiotic exposure, environment, and complementary feeding [11]. Microbial fermentation of dietary fibres and resistant starches produces metabolites such as short-chain fatty acids (SCFAs), which contribute to epithelial barrier integrity, immune regulation, and metabolic homeostasis [12,13]. Microbiome alterations have been associated with several maternal and childhood disorders, although causal relationships remain unresolved for some conditions [14]. Diet is also an important modulator of microbial composition and function [15,16]. These interactions provide the biological rationale for microbiome-informed maternal and child nutrition research, while their clinical and public health implications remain an emerging area of investigation.

### 1.2. Indigenous African Food Systems and the Evidence Gap

Growing evidence has increased interest in fermented foods, probiotics, postbiotics, and fibre-rich diets as potential microbiome-modulating strategies [17,18]. However, findings remain heterogeneous across strains, interventions, populations, and methodologies. Indigenous African food systems represent an under-investigated reservoir of dietary diversity, fermentation-associated microorganisms, bioactive compounds, and culturally embedded nutritional practices that may be relevant to microbiome–host interactions. Traditional foods and fermented products may provide fibre, resistant starches, bioactive compounds, and beneficial microorganisms [19]. However, evidence directly linking these food systems with maternal and child microbiome outcomes remains limited and heterogeneous. African populations are also underrepresented in microbiome research despite their diverse diets and substantial burden of maternal and child malnutrition [20]. This evidence gap strengthens the need for context-specific research examining indigenous foods and microbiome–nutrition interactions in African populations and for determining whether such foods could plausibly inform future nutrition interventions.

### 1.3. Evidence Gaps and Rationale for the Review

Important limitations remain in microbiome-informed nutrition research. Much evidence is observational and therefore provides limited evidence of causality [21]. Studies also frequently emphasize microbial composition rather than microbial function, with relatively fewer integrating multi-omics approaches [22]. Geographical and methodological disparities further limit generalizability and comparability [23]. Previous reviews have commonly focused on individual interventions, diseases, or microbial characteristics, with less integration of indigenous food systems, fermented foods, probiotics, postbiotics, microbial metabolites, mechanisms, and public health applications [24,25]. This review addresses this gap by integrating mechanistic, nutritional, microbiological, and public health evidence, with particular attention to indigenous African food systems and resource-constrained settings while distinguishing evidence-supported implications from proposed or hypothesis-generating applications.

### 1.4. Scope of the Review

This review synthesizes evidence on maternal–infant microbiome development, diet–microbiome interactions, indigenous food systems, traditional fermented foods, probiotics, postbiotics, microbial metabolites, and host–microbiome mechanisms. The review aims to critically examine how interactions among diet, the gut microbiota, microbial metabolites, and host responses may influence maternal and child health and to evaluate their potential translation into culturally appropriate nutrition and public health strategies as areas for future research rather than established implementation approaches. Importantly, the review distinguishes established evidence from emerging and hypothesis-generating applications. The MINF proposed later is therefore presented as a conceptual framework rather than a clinically validated model.

## 2. Methodology

### 2.1. Review Design and Scope

This study was conducted as a structured narrative review and purposive narrative evidence map of the literature on microbiome-informed maternal and child nutrition, with particular emphasis on indigenous African foods, fermented foods, probiotics, postbiotics, microbial metabolites, and their potential relevance to maternal and early-life health. A structured narrative approach was adopted because the review encompasses evidence from diverse populations, study designs, food systems, microbiological investigations, mechanistic studies, and emerging translational applications that cannot be adequately synthesized using a single intervention-based framework. The review was designed to provide a transparent, thematically organized synthesis and mapping of relevant evidence rather than to undertake a systematic or scoping review.

### 2.2. Literature Search Strategy

A comprehensive literature search was conducted in PubMed, Scopus, Web of Science, ScienceDirect, and Google Scholar, selected for their coverage of biomedical, nutritional, microbiological, food-science, and molecular research. The search focused primarily on publications from January 2015 to December 2025, with the final search conducted in June 2026. Search strategies combined controlled vocabulary, including Medical Subject Headings (MeSH) where applicable, and free-text terms using the Boolean operators AND and OR. Major search concepts included microbiome AND maternal nutrition, microbiome AND pregnancy, infant microbiome AND nutrition, indigenous African foods AND microbiome, fermented foods AND maternal nutrition, probiotics AND pregnancy, and postbiotics AND maternal-child health. Additional keywords included maternal–infant axis, dysbiosis, short-chain fatty acids, nutritional resilience, functional metabolites, immune homeostasis, precision nutrition, and early-life programming. Search terms were adapted to the indexing requirements of individual databases. Reference lists of relevant publications were also manually screened to identify additional literature. The search strategy was intended to identify a broad and relevant evidence base for purposive thematic mapping, rather than to establish an exhaustive systematic-review dataset.

### 2.3. Eligibility Criteria

Publications were considered for inclusion when they were published in English and addressed one or more aspects of microbiome-informed maternal and child nutrition, including gut microbiota, maternal or infant health, dysbiosis, indigenous or traditional food systems, fermented foods, probiotics, postbiotics, microbial metabolites, immune regulation, early-life programming, or precision nutrition. Both human and non-human evidence were considered, including clinical and observational studies, animal studies, in vitro investigations, and relevant systematic reviews, narrative reviews, and meta-analyses. Animal and in vitro studies were retained where they provided mechanistic or biological evidence relevant to the review objectives. Publications were not considered when they were outside the thematic scope of the review, lacked sufficient relevant information, or were editorials, commentaries, conference abstracts without adequate supporting information, or non-English publications.

### 2.4. Purposive Evidence Identification and Selection

Rather than applying a formal systematic-review screening procedure, literature was purposively identified and selected according to its relevance to the predefined objectives and thematic scope of the review. Retrieved publications were examined for their relevance to maternal and child nutrition, microbiome development and function, indigenous African food systems, fermented foods, probiotics and postbiotics, microbial metabolites, immune regulation, safety, and translational applications. Reference lists of key publications were also examined to identify additional relevant evidence. The selection process therefore emphasized conceptual relevance, population and study-design diversity, methodological characteristics, and directness of evidence to maternal and child nutrition, rather than numerical completeness or exhaustive database screening. This approach enabled the review to map established findings alongside emerging, mechanistic, and hypothesis-generating evidence across interconnected research areas.

### 2.5. Evidence Characterization and Narrative Synthesis

The identified literature was characterized according to population and study design, including (i) human randomized or intervention studies, (ii) prospective or longitudinal studies, (iii) cross-sectional and other observational studies, (iv) animal studies, (v) in vitro studies, and (vi) reviews and mechanistic literature. Methodological quality and scientific rigor were considered in relation to study design. For human studies, the assessment considered study design appropriateness, sample size where reported, participant selection, the presence of appropriate controls or comparators, the validity of microbiome and clinical outcome measurements, completeness of methodological reporting, and potential sources of bias. For animal and in vitro studies, experimental design, appropriate controls, methodological transparency, reproducibility, and biological relevance were considered. For reviews and meta-analyses, methodological transparency, quality of the underlying evidence, and consistency of findings were considered. These criteria were used to inform interpretation and weighting of evidence rather than to apply a formal numerical quality score These evidence characteristics were also used to structure the tables allowing differentiation of population, study design, microbiome endpoints, clinical or nutritional endpoints, and directness to maternal–child health for each evidence-based or proposed application. Evidence was then synthesized narratively and organized around maternal–infant microbiome development, microbiome–host interactions, indigenous African food systems, fermented foods, probiotics and postbiotics, microbial metabolites, immune regulation, translational applications, safety considerations, and future research priorities. Where evidence was derived from animal or in vitro studies, it was interpreted as preclinical or mechanistic evidence rather than direct clinical evidence. This purposive evidence-mapping approach was used to distinguish relatively established findings from emerging and hypothesis-generating evidence while avoiding claims of exhaustive systematic study identification.

## 3. Thematic and Mechanistic Analysis

### 3.1. Current Understanding and Concepts

#### 3.1.1. Development of the Maternal–Infant Microbiome

The maternal–infant microbiome develops through a dynamic continuum that begins before birth and continues throughout infancy, with potential implications for immune maturation, metabolic programming, and long-term health [26]. Although the concept of a resident placental microbiome remains controversial, accumulating evidence demonstrates that maternal physiological status, including diet, obesity, antibiotic exposure, inflammation, and gut microbial composition, may influence fetal development through maternal–fetal transfer of microbial metabolites, immune mediators, and nutrients [27]. Although maternal–fetal transfer of microbial metabolites, immune mediators, and nutrients is biologically plausible and supported by experimental evidence, evidence for a resident placental or fetal microbiome remains unresolved. Microbial signals in low-biomass prenatal samples may be influenced by reagent-derived contaminants (“kitome”) and environmental contamination during deoxyribonucleic acid (DNA) extraction, amplification, and sequencing. Therefore, detection of bacterial DNA alone should not be interpreted as evidence of viable in utero colonization without rigorous contamination controls and molecular validation [28]. Microbial-derived metabolites, such as SCFAs, can cross the placental barrier and may influence fetal immune differentiation, intestinal development, and epigenetic programming [29]. Consequently, maternal microbial health during pregnancy may represent one of several factors relevant to neonatal physiological development, though there is no established direct colonization of the fetal gut.

Birth represents the first major ecological transition in microbial establishment [30]. Vaginally delivered infants are initially exposed to microorganisms originating primarily from the maternal vaginal and intestinal microbiota, including *Lactobacillus* sp., *Bacteroides* sp., and *Bifidobacterium* sp. [31]. In contrast, infants delivered by cesarean section may initially acquire different microbial communities, including greater representation of skin- and healthcare-associated microorganisms such as *Staphylococcus* sp. and *Corynebacterium* sp. [32]. These differences are most pronounced during the early postnatal period and may influence microbial succession, immune maturation, and metabolic function. However, accumulating longitudinal studies suggest that postnatal factors including breastfeeding, complementary feeding, antibiotic exposure, and environmental contact substantially modify these early microbial differences, indicating that birth mode represents one of several determinants of microbiome development rather than its sole predictor [33,34].

Breastfeeding is widely recognized as one of the strongest modulators of early-life microbial maturation. Human breast milk provides not only essential nutrients but also a complex array of bioactive components, including human milk oligosaccharides (HMOs), immunoglobulins, antimicrobial peptides, cytokines, and commensal microorganisms [35]. HMOs selectively promote the growth of beneficial bacteria, particularly Bifidobacterium species, which metabolize these complex carbohydrates into SCFAs and other metabolites that can contribute to epithelial barrier integrity, immune regulation, and resistance to pathogen colonization [36]. Breastfeeding therefore supports the establishment of an early-life microbial ecosystem associated with immune education and intestinal development and has been associated with lower risks of diarrheal diseases, respiratory infections, allergies, and necrotizing enterocolitis [37].

The transition to complementary feeding introduces a broader diversity of dietary substrates that progressively reshape the infant gut microbiota toward a more adult-like configuration. Increased consumption of plant-derived fibers, resistant starches, proteins, and complex polysaccharides supports expansion of metabolically diverse bacterial communities capable of producing SCFAs and other bioactive metabolites. Conversely, inadequate dietary diversity, excessive consumption of ultra-processed foods, recurrent antibiotic exposure, and environmental enteric dysfunction have been associated with altered microbial maturation and may be associated with impaired nutrient utilization, immune dysregulation, and increased susceptibility to adverse health outcomes [38]. These developmental stages highlight the maternal–infant microbiome as a dynamic biological system that is highly responsive to nutritional and environmental influences during early life. The sequential establishment of the maternal–infant microbiome from pregnancy through early childhood is illustrated in Figure 1.

#### 3.1.2. Gut Dysbiosis and Disease

Gut dysbiosis is characterized by alterations in the composition, diversity, and functional capacity of the intestinal microbiota that may disrupt microbial homeostasis and host physiology [39]. Rather than reflecting the loss or gain of individual microbial species, dysbiosis can involve changes in microbial community structure and metabolic functions, including reduced production of SCFAs, impaired bile acid metabolism, altered vitamin synthesis, and increased generation of pro-inflammatory metabolites. These functional disturbances have been linked to epithelial barrier dysfunction, chronic immune activation, oxidative stress, and metabolic dysregulation and may provide biological pathways through which dysbiosis could influence maternal and child health [40].

In maternal and pediatric populations, dysbiosis has been associated with a broad spectrum of nutritional, metabolic, inflammatory, and infectious disorders [41]. Studies have reported associations between altered microbial composition and conditions including obesity, gestational diabetes mellitus, childhood undernutrition, inflammatory bowel disease, and recurrent enteric infections [42]. Although reduced abundance of some SCFA-producing taxa, including *Faecalibacterium* sp., *Roseburia* sp., and *Bifidobacterium* sp., has been reported in some disease states, microbial signatures vary substantially according to disease, age, diet, geography, and other environmental factors. Accordingly, these microbial patterns should be interpreted primarily as associations rather than universal disease-specific signatures [43].

Emerging evidence further indicates that gut dysbiosis may be involved in the biological processes associated with childhood stunting through mechanisms extending beyond inadequate dietary intake [44]. Children with severe acute malnutrition frequently exhibit delayed microbial maturation characterized by altered microbial diversity and depletion or delayed development of bacterial communities involved in carbohydrate fermentation and other metabolic functions. These alterations may affect nutrient bioavailability, intestinal barrier function, and immune competence and could potentially influence responsiveness to conventional nutritional rehabilitation [45]. Similar microbial disturbances have been implicated in obesity, allergic diseases, and type 2 diabetes, where altered microbial metabolite production may be involved in pathways related to energy metabolism, immune regulation, and endocrine signaling [46]. Although causal relationships remain under investigation, microbiome-directed nutritional interventions are increasingly being explored as potential complementary strategies for investigation alongside established approaches, rather than established treatments for disease prevention and management. Their potential role in maternal and child health therefore remains a hypothesis-generating area requiring further mechanistic, longitudinal, and intervention-based research. The associations between gut dysbiosis and major maternal and child health conditions are summarized in Table 1.

### 3.2. Mechanistic Insights

The potential health effects of microbiome-informed nutrition are mediated through a network of microbial metabolites, host signaling pathways, and immune responses that collectively regulate intestinal homeostasis and systemic physiology. Rather than acting solely through changes in microbial composition, dietary interventions including probiotics, fermented foods, and fiber-rich indigenous diets may influence microbial metabolic activity and the production of bioactive compounds that interact with epithelial, immune, and metabolic pathways [55]. These mechanisms provide biological plausibility for microbiome-targeted nutritional strategies, although mechanistic evidence does not necessarily establish clinical effectiveness in maternal and child populations.

#### 3.2.1. Probiotics and Postbiotics

Probiotics are defined as live microorganisms that confer health benefits when administered in adequate amounts [56,57], whereas postbiotics comprise preparations of inanimate microorganisms and/or their components that exert biological activity in the host [58]. Common probiotic genera include *Lactiplantibacillus* sp., *Lacticaseibacillus* sp., Bifidobacterium sp., *Leuconostoc* sp., and *Pediococcus* sp., many of which are naturally present in fermented foods. These microorganisms can contribute to microbial community interactions, colonization resistance, epithelial barrier function, immune modulation, and production of bioactive metabolites, although effects are often strain-, dose-, host-, and context-dependent [59]. Postbiotics may offer practical advantages related to stability and the absence of viable microorganisms, but their safety, efficacy, and optimal applications in maternal and child nutrition require population-specific evaluation [60]. Accordingly, the presence of probiotic or postbiotic properties in a microorganism or food matrix should not be interpreted as evidence of clinical benefit in maternal or child populations without appropriate intervention-based evidence.

#### 3.2.2. Short-Chain Fatty Acids

SCFAs are major metabolites generated by microbial fermentation of dietary fibers and resistant starches [61]. Acetate is the most abundant SCFA and participates in lipid metabolism, cholesterol synthesis, and peripheral energy utilization [62]. Propionate is transported to the liver, where it can contribute to metabolic processes and influence enteroendocrine signaling [63]. Butyrate serves as an important energy source for colonocytes and has been implicated in maintaining epithelial integrity, regulating inflammatory responses, and supporting regulatory T-cell functions [64]. Collectively, SCFAs activate G-protein-coupled receptors and inhibit histone deacetylases, thereby linking dietary intake with immune, metabolic, and epigenetic processes in experimental and physiological contexts.

#### 3.2.3. Molecular Mechanisms

The biological effects of microbial metabolites are mediated through interconnected metabolic and signaling pathways. SCFAs are synthesized through microbial enzymatic pathways, including the acetyl-CoA, succinate, and butyryl-CoA pathways, depending on substrate availability and microbial composition. These metabolites have been shown to influence intestinal barrier function through effects on epithelial cells and tight-junction regulation [65]. In parallel, microbial metabolites can modulate immune homeostasis, including regulatory T-cell differentiation and inflammatory cytokine responses [66]. At the cellular level, these responses involve signaling pathways including AMP-activated protein kinase (AMPK), mechanistic target of rapamycin (mTOR), mitogen-activated protein kinase (MAPK), nuclear factor-kappa B (NF-κB), Toll-like receptor (TLR), and peroxisome proliferator-activated receptor (PPAR) signaling [67]. Together, these pathways provide mechanistic links between microbial metabolism, host immunity, epithelial function, and energy metabolism, but their translation into effective maternal and child nutrition interventions remains an area for further investigation. These mechanistic pathways therefore provide a basis for hypothesis generation and experimental investigation rather than evidence of established clinical efficacy. The principal molecular pathways mediating microbiome–host interactions are highlighted in Table 2.

### 3.3. Indigenous African Foods, Traditional Dietary Patterns and Fermented Foods

#### 3.3.1. Indigenous African Foods and Dietary Substrates

Indigenous African food systems represent an under-investigated reservoir of nutrient-dense legumes, dietary fiber, proteins and essential amino acids, polyunsaturated fatty acids, micronutrients, and bioactive compounds that may support food security, nutritional health, and gut microbial ecology [82]. Traditional foods such as millet, sorghum, acha/fonio, Bambara groundnut, cowpea, African leafy vegetables, and other locally available plant foods provide substrates that can be metabolized by intestinal microorganisms and may contribute to microbial diversity and metabolite production [83]. Millet contains dietary fiber, resistant starch, phenolic acids, and flavonoids, while sorghum provides resistant starch and polyphenolic compounds that may influence microbial fermentation and metabolic activity [84]. Cowpea and African leafy vegetables similarly provide fiber, oligosaccharides, polyphenols, carotenoids, and other micronutrients that may support dietary quality and intestinal microbial metabolism [85]. Importantly, the available evidence varies considerably in strength. Human dietary studies and experimental fermentation studies provide evidence for some foods, whereas for others the microbiome-related effects remain largely inferred from nutritional composition, in vitro studies, or animal models. Therefore, these foods should be considered promising dietary substrates rather than established microbiome-directed interventions. This distinction is particularly important in maternal and child nutrition, where dietary adequacy, cultural acceptability, food availability, and food safety must be considered alongside the potential for microbiome effects.

#### 3.3.2. Traditional Fermented Foods and Microbial Diversity

Traditional fermentation is an important component of African food systems, supporting food preservation, food security, and nutritional quality while generating bioactive compounds such as vitamins, peptides, and short-chain fatty acids through diverse microbial communities, particularly lactic acid bacteria, yeasts, and molds [86,87]. Fermentation drives biochemical transformations that can affect food safety, nutrient availability, and fiber fermentability, while generating bioactive metabolites such as SCFAs, peptides, exopolysaccharides, bacteriocins, and vitamins. These microbial products may influence gut microbial diversity, barrier function, immune regulation, and metabolic health, although outcomes vary with fermentation methods, microbial composition, and individual responses [88,89,90]. Traditional fermented foods are of major importance to food security, nutrition, health, and socio-economic development in Africa. Products such as ogi, kunu, nono, and dawadawa (iru) represent diverse cereal-, dairy-, and legume-based fermentations, in which microbial communities, particularly lactic acid bacteria and yeasts, contribute to preservation, nutritional transformation, and the production of bioactive metabolites. Recent microbiome studies have further revealed diverse microbial populations with potential technological, nutritional, and health-related applications [91]. However, the presence of potentially beneficial microorganisms in fermented foods should not automatically be interpreted as evidence of probiotic efficacy in humans. Probiotic effects are strain-specific and depend on viability, dose, food matrix, host characteristics, and demonstrated health outcomes. Most evidence for traditional African fermented foods currently comes from food microbiology, fermentation studies, compositional analyses, and experimental research rather than maternal or pediatric clinical trials. Accordingly, traditional fermented foods should be viewed as potential microbiome-modulating foods and sources of candidate microorganisms, rather than as clinically validated probiotic interventions.

#### 3.3.3. Nutritional and Microbiome-Modulating Potential

The relevance of indigenous foods to microbiome-informed nutrition lies in the interplay between their nutritional composition, fermentation processes, microbial diversity, and cultural acceptability, with potential implications for food safety and human health [92]. Fiber-rich grains, legumes, vegetables, and fermented foods provide nutrients and substrates that shape gut microbiota and microbial metabolite production, although the extent and clinical significance of these effects vary across foods and populations [93]. The evidence summarized in Table 3 and Table 4 illustrates this diversity, while the complex interactions between fermented foods and the gut microbial ecosystem are depicted in Figure 2. Plant-based foods primarily contribute fermentable carbohydrates, resistant starch, fiber, polyphenols, and micronutrients, whereas fermented foods additionally provide microorganisms and fermentation-derived metabolites. Together, these characteristics provide a biologically plausible basis for investigating indigenous food systems as components of microbiome-informed maternal and child nutrition. Nevertheless, evidence should not be interpreted as demonstrating that consumption of individual indigenous foods directly prevents malnutrition, dysbiosis, or disease. Rather, current findings support their investigation as culturally appropriate dietary resources whose microbiome-related effects require further human intervention studies. This distinction is particularly relevant for African populations, where food systems, dietary practices, environmental exposures, and microbiome composition may differ substantially from those of populations represented in existing microbiome research. Population-specific studies are therefore needed to determine whether microbiome responses to indigenous foods are reproducible and clinically meaningful and whether such responses translate into measurable maternal or child health outcomes.

Evidence interpretation: Direct evidence refers to studies conducted in relevant maternal or child populations with corresponding microbiome and/or clinical or nutritional outcomes. Indirect evidence refers to studies conducted in other populations or settings that may inform maternal and child nutrition but do not directly establish maternal–child effects. Food microbiology, in vitro, and animal studies are considered mechanistic or preclinical evidence and are interpreted as hypothesis-generating rather than as direct clinical evidence. “Not assessed” indicates that the relevant microbiome or clinical endpoint was not measured in the cited study. Potential health or translational relevance should not be interpreted as established clinical benefit.

### 3.4. Fermented Foods, Probiotics and Postbiotics

Fermented foods bridge traditional food systems and microbiome-informed nutrition, while their health benefits, safety, and regulatory frameworks require further mechanistic and clinical evidence [110]. Traditional African fermented foods harbour diverse lactic acid bacteria that drive fermentation and contribute to food safety, shelf life, nutritional quality, and potential health benefits through bioactive metabolite production [111]. The presence of probiotic candidate microorganisms in a fermented food does not by itself establish that the food is a probiotic intervention. Demonstration of a probiotic effect requires evidence for an adequately characterized strain, appropriate dose, viability where relevant, and a defined health benefit [112]. For maternal and child nutrition, the most immediate evidence-supported role of fermented foods is therefore their contribution to food preservation, digestibility, nutrient availability, and dietary diversity, while their potential microbiome-modulating and probiotic effects remain areas for further investigation. Postbiotics offer promising microbiome-targeted benefits with reduced risks associated with live microorganisms, although their efficacy and safety require further study [113]. Nevertheless, clinical evidence for postbiotic applications specifically in maternal and child nutrition remains limited, and safety, formulation, dose, and population-specific efficacy require further evaluation. Thus, fermented foods, probiotics, and postbiotics are positioned in this review along an evidence continuum: fermented foods are established components of traditional diets; selected microorganisms isolated from these foods may represent candidates for probiotic development; and postbiotic preparations represent an emerging intervention category requiring further clinical validation.

### 3.5. Microbiome-Mediated Nutritional and Health Implications

Maternal and child nutrition influences early-life gut microbiome development, immune and metabolic function, and long-term health, offering a basis for investigating microbiome-targeted interventions. Evidence from African and other low-resource settings provides important context for understanding how these interactions may influence maternal and child nutritional and health outcomes, although the available evidence remains heterogeneous and comparatively limited [114]. Most microbiome research has been conducted in Europe, North America, and East Asia, resulting in limited representation of African populations despite their exceptional dietary diversity, environmental heterogeneity, and distinct microbial exposures. Existing African studies indicate that gut microbiota composition is influenced by traditional dietary patterns, breastfeeding practices, infectious disease burden, sanitation, antibiotic exposure, and urbanization [115]. Compared with populations consuming Westernized diets, rural African communities often exhibit greater microbial diversity and higher abundance of fiber-degrading microorganisms associated with increased production of SCFAs [116]. However, rapid nutrition transition and changing food systems are increasingly altering these microbial profiles, with potential implications for maternal and child health.

#### 3.5.1. Evidence from Africa and Low-Resource Settings

Maternal and child nutrition studies from Africa have primarily focused on childhood stunting, severe acute malnutrition, environmental enteric dysfunction, breastfeeding, and complementary feeding [117]. Emerging evidence suggests that microbial immaturity, reduced abundance of beneficial fermentative bacteria, and impaired microbial metabolic capacity may be associated with poor growth, immune dysfunction, and reduced response to nutritional rehabilitation [118]. However, these associations should not be interpreted as evidence of direct causality, as diet, infection, sanitation, socioeconomic conditions, and other environmental exposures may also influence both microbiome development and nutritional outcomes. Nevertheless, relatively few studies have integrated microbiome profiling with indigenous diets, fermented foods, metabolomics, or longitudinal maternal–infant cohorts, limiting understanding of region-specific microbiome–nutrition interactions [119]. This evidence gap is particularly important in Africa, where indigenous food systems represent an under-investigated reservoir of dietary diversity, fermentation-associated microorganisms, bioactive metabolites, and culturally embedded nutritional practices. Scientific investigation of these food systems may help identify culturally appropriate and affordable nutritional strategies, but their microbiome-mediated health effects require further validation through well-designed human studies. Future studies should prioritize multidisciplinary collaborations integrating food science, microbiology, implementation science, and public health, with greater emphasis on longitudinal maternal–infant cohorts, well-characterized dietary exposures, microbiome profiling, metabolomics, and intervention studies. Such research may help determine whether findings from indigenous food systems can be translated into context-appropriate nutritional approaches. 

#### 3.5.2. Maternal and Child Nutritional Implications

The available African evidence suggests that microbiome-related nutritional pathways may be relevant to breastfeeding, complementary feeding, growth, environmental enteric dysfunction, and metabolic health, but the strength of evidence varies considerably across outcomes. In rural Malawi, selected environmental exposures were not significantly associated with environmental enteric dysfunction biomarkers (EED) biomarkers among 18-month-old children, suggesting context-specific drivers of EED [120]. These findings highlight the importance of distinguishing associations between microbiome-related factors and nutritional outcomes from demonstrated causal effects. Evidence from Abu Dhabi highlights gaps in complementary feeding, with only 36.2% of children meeting the minimum acceptable diet despite timely introduction and adequate dietary diversity in many children [121]. Commercial plant-based infant foods vary nutritionally, highlighting the need for complete labelling and informed parental choices regarding protein, micronutrients, sugar, and other nutrients [122]. Together, these findings highlight the relevance of nutrition education that promotes dietary diversity and the appropriate use of locally available nutrient-dense foods rather than reliance on a single food category. Population studies show that gut microbiome composition varies with geography and lifestyle, underscoring the need for greater representation of African populations in microbiome research and population-specific nutrition strategies [123]. Traditional fermentation in Nigeria transforms locally available crops such as maize, cassava, millet, sorghum, yam, and nuts into diverse foods, improving digestibility, nutritional quality, sensory properties, shelf life, and potential health benefits [124]. However, the potential nutritional and microbiome benefits of these foods should be distinguished from demonstrated clinical outcomes, which remain insufficiently studied in maternal and child populations.

#### 3.5.3. Translational and Public Health Implications

Taken together, the evidence supports the potential relevance of microbiome-informed nutrition to maternal and child health, while also demonstrating that translation into clinical and community practice remains at an early stage. Indigenous foods, breastfeeding promotion, dietary diversity, fermented foods, and locally appropriate food-processing practices may warrant investigation as potential entry points for future nutrition interventions in low-resource settings. However, microbiome-based recommendations should be considered alongside, rather than as replacements for, established maternal and child nutrition interventions. Future translational research should therefore determine whether microbiome-informed dietary strategies produce measurable improvements in nutritional status, growth, metabolic health, immune function, or other clinically meaningful outcomes. Such studies should also evaluate safety, acceptability, cultural appropriateness, feasibility, and sustainability before any widespread implementation. Table 5 outlines representative microbiome and maternal–child nutrition studies from Africa, a selection of relevant African studies is provided to inform maternal and child nutrition.

### 3.6. Community-Based and Primary Healthcare Applications

Translation of microbiome science into maternal and child health requires interventions that extend beyond clinical settings into communities and primary healthcare systems. Food-based microbiome interventions may be of particular interest for investigation in low-resource settings because they build upon existing dietary practices rather than relying exclusively on commercial probiotic formulations [131]. Potentially integrating microbiome-informed nutrition into antenatal care, infant feeding programs, community health outreach, and nutrition counseling could be explored as an adjunct to established nutrition interventions, although the effectiveness of microbiome-informed approaches in routine maternal and child healthcare remains insufficiently validated [132]. Community nutrition education represents a potential implementation strategy for investigation. Educational programs could potentially promote the consumption of fiber-rich indigenous foods, appropriately and safely prepared traditional fermented foods, exclusive breastfeeding, and age-appropriate complementary feeding, while discouraging excessive intake of ultra-processed foods [133]. Training community nutrition champions, community health workers, and primary healthcare personnel could potentially support the dissemination of evidence-based nutrition messages and explore the acceptability of microbiome-informed dietary interventions within local populations [134]. Such approaches should emphasize established nutrition and food-safety recommendations while avoiding unvalidated claims about specific microbiome benefits. Before broader implementation is considered, supportive policies would require relevant microbiome evidence, where sufficiently established, to be evaluated for integration into national nutrition strategies, food-based dietary guidelines, maternal and child health programs, and agricultural policies. Strengthening collaborations among researchers, clinicians, nutritionists, policymakers, and local communities could support research to determine whether emerging microbiome evidence can be translated into sustainable public health interventions that are culturally appropriate, economically feasible, and scalable across diverse African settings. Importantly, community implementation research should incorporate food-safety safeguards. Traditional fermentation does not inherently guarantee microbiological safety, and safety may vary according to ingredients, fermentation conditions, processing practices, storage, and duration. Particular attention is required when fermented foods or microbiome-targeted products are intended for pregnant women, infants, young children, or other vulnerable populations. Table 6 summarizes potential community-based applications of microbiome-informed maternal and child nutrition, highlighting how indigenous foods, fermented foods, dietary education, and primary healthcare partnerships could potentially be investigated as components of context-specific community interventions. A conceptual framework for integrating microbiome science into community healthcare systems is presented in Figure 3. Current evidence supports the potential of community and primary healthcare platforms to deliver nutrition interventions, but direct evidence that microbiome-informed approaches improve maternal or child clinical outcomes remains limited. Strengthening multidisciplinary research, validating indigenous food systems, and evaluating whether appropriately supported microbiome science can be integrated into community nutrition programs and primary healthcare may provide a basis for future translation, rather than establishing current pathways to scalable and sustainable public health implementation in low-resource settings.

### 3.7. Emerging Technologies and Innovations

Rapid advances in computational biology and high-throughput molecular technologies are transforming microbiome research from descriptive microbial profiling toward functional characterization and data-driven investigation of diet–microbiome–host interactions [144]. These innovations enable researchers to identify microbial functions, host–microbe interactions, and metabolic pathways that may influence maternal and child health, thereby supporting research into potential precision microbiome-based nutritional approaches [145]. These technologies are improving understanding of how dietary exposures, microbial communities, and host physiology interact across pregnancy, infancy, and early childhood. Artificial intelligence (AI) has emerged as a powerful tool for integrating complex microbiome datasets with dietary, clinical, genetic, and environmental information [146]. Machine learning algorithms are increasingly being used to predict disease risk, identify microbial biomarkers, classify microbial communities, and investigate potential relationships between microbial characteristics and dietary or clinical phenotypes [147]. Although most AI-driven microbiome applications remain in the research domain, they may support future applications in nutritional assessment, disease-risk prediction, and potentially more individualized approaches to maternal and child nutrition. Multi-omics technologies have further expanded the mechanistic understanding of microbiome–host interactions [148]. Metagenomics characterizes microbial taxonomic composition and functional genes, metabolomics identifies microbial metabolites involved in host physiology, transcriptomics examines gene expression responses, and proteomics evaluates functional proteins involved in microbial and host metabolic processes [149]. Integration of these complementary datasets provides a systems-level understanding of microbial function that extends beyond taxonomic profiling and may facilitate the identification of candidate biomarkers and pathways for future intervention research. Emerging approaches, including microbiome engineering and precision microbiome nutrition, aim to deliberately modify microbial ecosystems through dietary interventions, targeted probiotics, engineered microbial consortia, postbiotics, and personalized nutrition strategies [150]. While these approaches remain in the early stages of clinical translation, they provide opportunities for research into individualized interventions based on microbiome composition, dietary patterns, and metabolic characteristics. However, these approaches should currently be regarded primarily as research and translational development strategies rather than as established maternal and child nutrition interventions. Broader implementation of validated applications will require standardized analytical methods, larger longitudinal cohorts, cost-effective technologies, independent validation, assessment of safety and clinical utility, and validation across diverse populations. Recent technological advances supporting microbiome-informed maternal and child nutrition are listed in Table 7.

### 3.8. Safety, Regulatory and Policy Considerations

The rapid expansion of microbiome-based nutritional interventions has highlighted the need for robust regulatory frameworks to ensure product quality, safety, efficacy, and appropriate clinical application. Regulatory oversight is particularly important for probiotics, postbiotics, microbiome-derived therapeutics, and functional foods intended for pregnant women, infants, and young children, who represent vulnerable populations requiring rigorous safety evaluation [159]. Safety considerations should extend beyond the biological identity of an intervention to include microbial contamination, the presence of pathogenic organisms, product consistency, strain identification, dose, strain-specific effects, antimicrobial resistance characteristics where relevant, food processing and storage conditions, and the intended population. For probiotics and postbiotics, safety assessment should therefore be considered across four key domains: microbial or product identity and quality, contamination and infection risk, dose- and strain- or preparation-specific effects, and population-specific susceptibility. These considerations are particularly important for traditionally fermented foods because microbial composition and safety can vary according to raw materials, fermentation practices, environmental conditions, processing, and storage. Fermented products containing alcohol also require particular caution in maternal and child nutrition, and alcohol-containing products should not be promoted as routine foods for pregnant women or children. For probiotic interventions, safety assessment should consider the identity and characteristics of the specific strain rather than assuming that safety demonstrated for one strain applies to another strain of the same species. Dose, duration of administration, host characteristics, underlying vulnerability, and potential antimicrobial-resistance determinants may also influence risk. Postbiotics may mitigate some risks associated with administering viable microorganisms, but their safety and biological effects still depend on the specific preparation, composition, dose, and target population. International organizations, including the World Health Organization (WHO) and the Food and Agriculture Organization (FAO), emphasize healthy diets, breastfeeding, food safety, and sustainable food systems and support evidence-based approaches to nutrition and food-related interventions. Regulatory agencies such as the United States Food and Drug Administration (FDA) and the European Food Safety Authority (EFSA) provide frameworks relevant to the evaluation of foods, probiotics, novel foods, health claims, manufacturing standards, and clinical evidence [160]. However, considerable variation exists among regulatory jurisdictions regarding product classification, efficacy requirements, strain identification, and health-claim authorization. For low- and middle-income countries, policy development should extend beyond regulation of commercial products to include consideration of indigenous food systems, traditional fermented foods, microbiome-informed dietary guidelines, and nutrition-sensitive agricultural policies [161]. However, incorporation of microbiome-specific recommendations into national guidelines should be proportionate to the strength of available evidence and should not precede adequate validation of safety, efficacy, feasibility, and acceptability. Strengthening national regulatory capacity, harmonizing standards, supporting local microbiome research, and establishing the evidence base needed to support validated microbiome applications in maternal and child nutrition programs may facilitate evidence-based implementation while supporting equitable access to safe and effective interventions. The contributions of major international organizations to microbiome-informed nutrition are summarized in Table 8.

### 3.9. Additional Safety Considerations for Vulnerable Populations

Pregnant women, preterm infants, neonates, young children, and severely malnourished individuals may require additional safeguards because altered immunity, intestinal barrier dysfunction, prematurity, or severe nutritional compromise may modify susceptibility to microbial or foodborne hazards. Accordingly, microbiome-targeted interventions intended for these groups should undergo population-specific safety assessment rather than relying exclusively on evidence obtained from healthy adults. For community-based fermented foods, implementation should therefore include appropriate raw-material selection, hygienic preparation, controlled or well-characterized fermentation where feasible, prevention of cross-contamination, appropriate storage conditions, and monitoring for spoilage or pathogenic contamination. Where standardized starter cultures are used, strain identity, functional characteristics, safety profile, and consistency should be documented.

Current evidence indicates that microbiome-informed maternal and child nutrition is a promising but still developing field rather than an established clinical paradigm. Advances in artificial intelligence, multi-omics technologies, precision nutrition, and microbiome engineering are providing new opportunities to investigate host–microbe interactions and explore more targeted nutritional interventions. However, significant challenges remain, including limited longitudinal and intervention studies, underrepresentation of African populations, methodological heterogeneity, limited clinical validation, safety uncertainties for vulnerable populations, and barriers to implementation in low-resource settings. Addressing these gaps through multidisciplinary research, standardized methods, appropriate safety evaluations, harmonized regulatory approaches, and careful evaluation of potential approaches for integration into community and primary healthcare programs will be essential to translating emerging discoveries into safe, equitable, culturally appropriate, and sustainable maternal and child nutrition strategies.

## 4. Innovations, Challenges, Limitations, and Future Perspectives

### 4.1. Innovations and Author Perspectives

Current evidence provides a basis for exploring a transition from conventional nutrient-centered maternal and child nutrition to microbiome-informed nutrition, in which dietary interventions could be investigated not only for their ability to meet nutritional requirements but also for their potential to influence microbial composition and function [168,169]. Based on the evidence synthesized in this review, we propose the Microbiome-Informed Nutrition Framework (MINF) as a conceptual and hypothesis-generating framework that integrates dietary, microbial, clinical, and public health components into a unified research framework for investigating potential approaches to maternal and child health. The framework is not presented as a clinically validated or implementation-tested intervention model, but rather as a conceptual structure for integrating evidence and generating testable hypotheses for future research. Unlike existing approaches that focus primarily on probiotic supplementation or individual dietary interventions, the proposed framework recognizes the gut microbiome as an important biological interface linking food systems, microbial metabolism, immune regulation, and health-related processes.

The MINF is organized into two interconnected domains: (A) evidence-supported biological and nutritional components and (B) proposed translational and implementation components. The evidence-supported components include maternal and child dietary exposures, dietary substrates, microbial composition and function, microbial metabolites, and host–microbiome interactions. The evidence-supported domain draws primarily on findings from nutritional, mechanistic, observational, and clinical intervention studies, whereas the translational and implementation domain represents author-proposed applications that require prospective clinical and implementation validation [170]. MINF differs from conventional nutrition and public-health frameworks by explicitly integrating dietary substrates, microbial ecology and function, microbial metabolites, and host–microbiome interactions with proposed translational and community-level applications within a single microbiome-informed framework. The MINF is founded on indigenous food systems, emphasizing the use of locally available whole grains, legumes, vegetables, fruits, and traditional fermented foods as sustainable sources of prebiotic substrates and, in some cases, candidate microorganisms with potential functional relevance. These dietary resources interact with the gut microbiota to influence microbial diversity, stimulate production of short-chain fatty acids and other bioactive metabolites, and participate in processes related to epithelial barrier function and immune homeostasis [171,172]. Within this framework, probiotics and postbiotics serve as complementary areas for investigation or potential interventions where evidence supports their clinical application, particularly among vulnerable maternal and pediatric populations. Their effects, however, may be strain-, formulation-, dose-, population-, and context-dependent and should therefore not be assumed to be uniform across populations.

A distinguishing feature of the proposed framework is the integration of potential translational applications with community-based implementation as areas for future research. While individualized nutrition based on microbiome characteristics represents a long-term objective, established public health measures such as primary healthcare systems, nutrition education, breastfeeding promotion, community nutrition champions, and culturally appropriate dietary recommendations provide existing platforms through which potential microbiome-informed approaches could be investigated [173]. Other proposed translational components include digital health, precision nutrition, and policy integration. These components represent potential applications requiring prospective evaluation rather than validated components of the MINF. Accordingly, the framework provides a conceptual bridge for investigating how emerging microbiome science might inform context-specific interventions, rather than establishing a pathway for immediate implementation or scale-up. The MINF proposed in this review is a conceptual and hypothesis-generating framework rather than a clinically validated intervention model. Its components are informed by evidence from nutritional, microbiome, mechanistic, observational, and intervention studies, but the framework itself has not undergone prospective clinical validation, stakeholder validation, or implementation testing. Future research should therefore evaluate its feasibility, acceptability, predictive utility, and clinical effectiveness in appropriately designed maternal and child nutrition studies. The proposed MINF, integrating evidence, community practice, and policy, is illustrated in Figure 4.

### 4.2. Emerging Innovations

Rapid technological advances are expanding opportunities for microbiome-informed maternal and child nutrition beyond conventional dietary interventions. Artificial intelligence and machine learning are increasingly being applied to analyze complex microbiome datasets, identify microbial biomarkers, predict disease susceptibility, and explore the potential for individualized dietary recommendations. When integrated with electronic health records and digital dietary assessment tools, these approaches may facilitate nutritional risk identification and support research into more personalized intervention strategies, although their clinical utility in maternal and child nutrition remains insufficiently validated [174].

Simultaneously, multi-omics technologies, including metagenomics, metabolomics, transcriptomics, and proteomics, are providing a systems-level understanding of microbial function by linking microbial composition to metabolic activity and host responses. These technologies enable the identification of functional biomarkers and molecular pathways that may inform precision nutrition and therapeutic development [175]. The emergence of postbiotic therapeutics, consisting of microbial metabolites or inactivated microbial preparations, also offers potential advantages over live probiotics, including greater stability and opportunities for more standardized production, although safety and clinical effectiveness remain dependent on the specific preparation and application [176].

Digital health technologies, including mobile applications, wearable devices, telehealth platforms, and artificial intelligence-assisted decision support systems, further create opportunities to investigate approaches for dietary monitoring, nutrition counseling, and potential delivery of microbiome-informed approaches within primary healthcare [177]. These technologies represent potential translational tools, but their effectiveness, accessibility, affordability, and suitability for maternal and child nutrition require evaluation in diverse populations and healthcare settings. Emerging innovations should therefore be regarded primarily as potential applications and research directions rather than established clinical interventions. Emerging innovations with potential applications in microbiome-informed maternal and child nutrition are presented in Table 9.

### 4.3. Challenges

Despite substantial progress, several challenges continue to limit the translation of microbiome science into routine maternal and child healthcare. Considerable inter-individual variability in microbiome composition, influenced by genetics, age, diet, geography, antibiotic exposure, and environmental factors, complicates the development of universally applicable nutritional recommendations. This variability underscores the importance of population-specific research and cautious interpretation of findings derived predominantly from high-income settings [184].

A second challenge is the limited representation of African and other low-resource populations within microbiome research. Most available datasets originate from Europe, North America, and East Asia, restricting the generalizability of current evidence to populations with different dietary patterns, environmental exposures, and disease burdens [185]. Furthermore, many studies are cross-sectional and involve relatively small sample sizes, limiting the ability to establish causal relationships or evaluate long-term microbiome dynamics.

Methodological heterogeneity also remains a significant barrier. Differences in sample collection, sequencing platforms, bioinformatic pipelines, microbial taxonomic classification, and outcome measures reduce comparability across studies [186]. In addition, lack of standardization in probiotic strains, postbiotic formulations, dosage regimens, and intervention durations complicates evidence synthesis and limits assessment of clinical effectiveness and comparability across interventions [187]. Although promising findings continue to emerge, well-designed, adequately powered randomized controlled trials evaluating microbiome-targeted interventions during pregnancy and early childhood remain limited.

### 4.4. Limitations

The interpretation of the current evidence should take into account several limitations in the published literature. Many studies involve modest sample sizes, short follow-up periods, and observational or cross-sectional designs that limit causal inference. Geographic bias remains evident, with disproportionate representation of high-income countries and relatively few longitudinal investigations from Africa and other low- and middle-income regions. Moreover, inconsistencies in sequencing methodologies, microbial classification systems, and analytical pipelines contribute to variability in reported microbial associations and reduce opportunities for direct comparison across studies. This review also has inherent limitations. Only English-language publications were included, potentially excluding relevant evidence published in other languages. As with most narrative reviews, the synthesis may also be influenced by publication bias, whereby studies reporting positive or statistically significant findings are more likely to be published. In addition, the heterogeneity of study populations, interventions, microbiome analytical methods, and reported outcomes limits direct comparison across studies and prevents firm conclusions regarding clinical effectiveness of microbiome-informed interventions. Nevertheless, by integrating evidence from diverse disciplines and emphasizing both mechanistic and translational perspectives, this review provides a broad synthesis of the current evidence on microbiome-informed maternal and child nutrition.

### 4.5. Future Perspectives

Future research should move beyond descriptive characterization of microbial communities toward functional and translational investigations that directly inform the development and evaluation of nutritional interventions. Particular priority should be given to the identification and characterization of indigenous probiotic strains isolated from traditional fermented foods, as these microorganisms may provide culturally acceptable and locally sustainable candidates for further investigation, rather than assuming equivalence with commercially available probiotic products [188].

Large longitudinal birth cohorts that incorporate maternal dietary assessment, microbiome profiling, metabolomics, and long-term child health outcomes are required to better characterize temporal relationships and to investigate potential causal pathways between microbial development and disease risk. Integration of multi-omics technologies with advanced bioinformatics will facilitate the identification of microbial biomarkers, functional metabolites, and signaling pathways that can inform future precision nutrition research [189].

Artificial intelligence and machine learning are expected to play an increasingly important role in integrating dietary, microbiome, genetic, clinical, and environmental data to investigate potential approaches to disease prediction and individualized nutritional recommendations. However, these applications require rigorous validation, representative datasets, assessment of algorithmic performance, and attention to data privacy and health equity before routine clinical implementation. At the same time, adequately powered multicentre randomized clinical trials are needed to evaluate the effectiveness, safety, and cost-effectiveness of probiotics, postbiotics, fermented foods, and indigenous dietary interventions across diverse populations [190].

Finally, future microbiome research should adopt a One Health perspective that recognizes the interconnected roles of human health, food systems, agriculture, environmental microbiology, and ecosystem sustainability [191]. Community-based interventions delivered through primary healthcare, supported by culturally appropriate nutrition education and evidence-based policy, may provide potential pathways for future investigation into translating microbiome science into equitable and sustainable maternal and child health strategies, but their feasibility, acceptability, effectiveness, and scalability should be evaluated through implementation research.

## 5. Conclusions

Maternal and child nutrition is increasingly understood as an interaction between diet, the gut microbiota, host metabolism, immune function, and environmental exposures. This review highlights the potential roles of indigenous foods, traditional fermented foods, probiotics, and postbiotics in influencing microbial composition, microbial metabolites, gut barrier function, and immune regulation, particularly within African and other resource-constrained settings. Indigenous and traditional fermented foods represent plausible and important research priorities because of their nutritional, cultural, and microbiome-relevant characteristics; however, they should not yet be considered proven microbiome interventions for maternal or child clinical outcomes. The proposed Microbiome-Informed Nutrition Framework (MINF) provides a conceptual and hypothesis-generating model that links evidence-supported relationships among dietary substrates, microbial ecology, microbial metabolites, and host–microbiome interactions to proposed translational applications, including precision nutrition, digital health, community nutrition, primary healthcare, and policy integration. MINF is not yet a validated intervention framework, and its feasibility, acceptability, predictive utility, and clinical effectiveness require prospective evaluation. Future research should prioritize longitudinal maternal–infant studies, multicentre intervention trials, standardized microbiome approaches, multi-omics integration, biomarker discovery, and investigation of indigenous probiotic resources. Rigorous prospective research is particularly needed to determine whether the microbiome-related properties of indigenous and fermented foods translate into measurable maternal and child health benefits. Such evidence will be essential for determining whether and how microbiome science can be translated into sustainable, equitable, and culturally relevant maternal and child nutrition strategies, particularly in Africa.

## Figures and Tables

**Figure 1:**
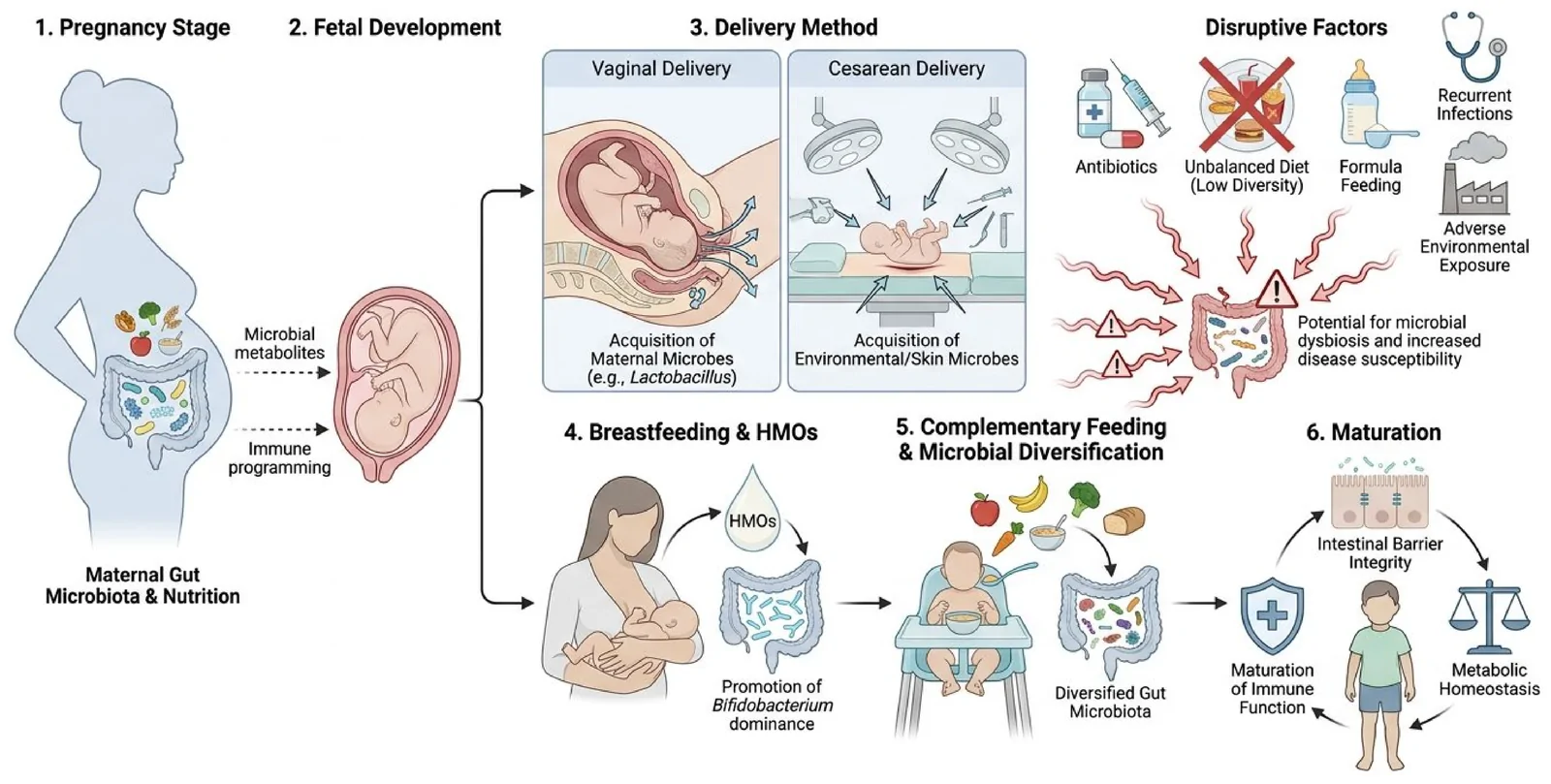
Development of the Maternal–Infant Microbiome (Created with FigureLabs). Schematic representation of maternal–infant microbiome development from pregnancy through early childhood, illustrating the sequential influence of (1) maternal nutritional status and gut microbiota during pregnancy, (2) microbial metabolite transfer and fetal immune programming, (3) microbial acquisition during vaginal or cesarean delivery, (4) breastfeeding and human milk oligosaccharides (HMOs) promoting *Bifidobacterium* sp. dominance, (5) complementary feeding and microbial diversification, and (6) maturation of immune function, intestinal barrier integrity, and metabolic homeostasis. The figure also highlights factors that disrupt normal microbial development, including antibiotic exposure, poor dietary diversity, formula feeding, recurrent infections, and adverse environmental exposures, which may contribute to microbial dysbiosis and increased susceptibility to disease.

**Figure 2:**
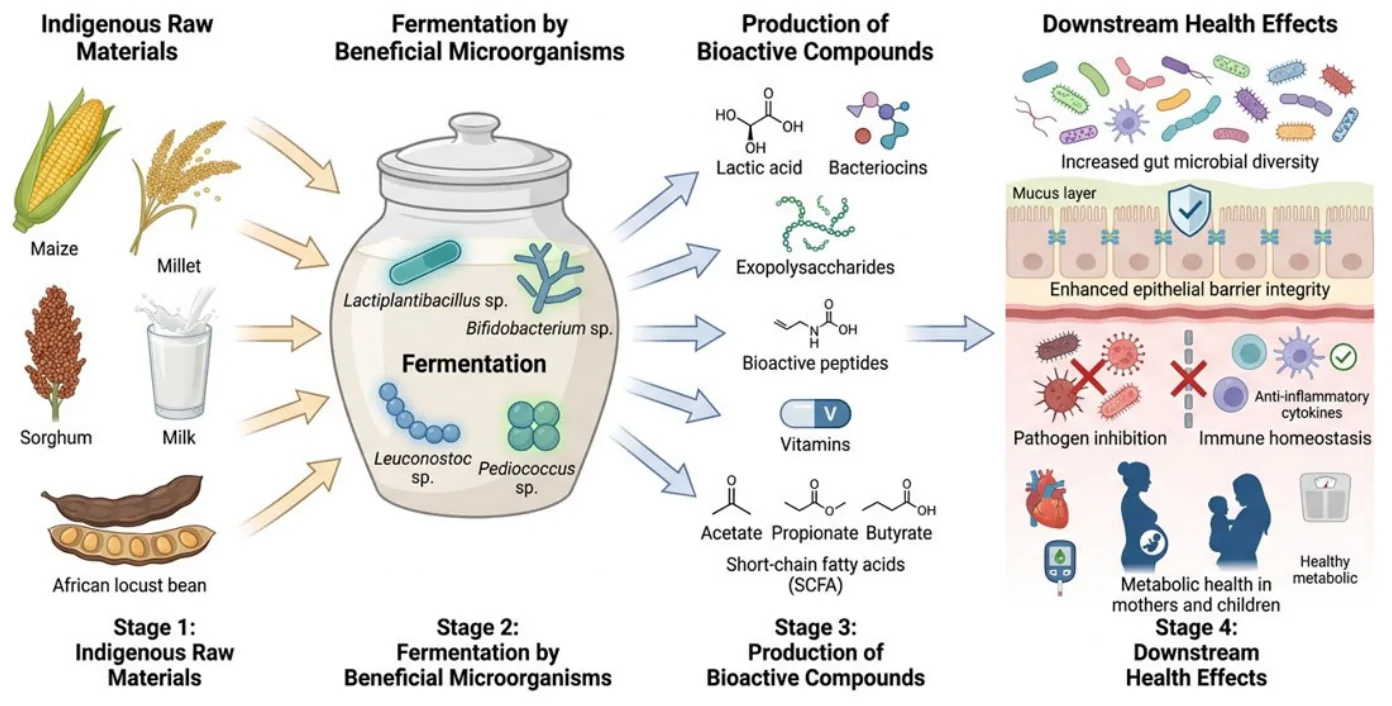
Fermented Foods and Gut Microbial Interactions (Created with FigureLabs). Schematic illustration of the interactions between traditional fermented foods and the gut microbiota, illustrating (i) indigenous raw materials, including maize, millet, sorghum, milk, and African locust bean; (ii) fermentation by beneficial microorganisms such as *Lactiplantibacillus* sp., *Bifidobacterium* sp., *Leuconostoc* sp., and *Pediococcus* sp.; (iii) production of bioactive compounds, including lactic acid, bacteriocins, exopolysaccharides, bioactive peptides, vitamins, and SCFAs; and (iv) their downstream effects on gut microbial diversity, epithelial barrier integrity, pathogen inhibition, immune homeostasis, and metabolic health in mothers and children. The figure highlights the role of traditionally fermented foods in modulating the gut microbiome and promoting maternal and child health.

**Figure 3:**
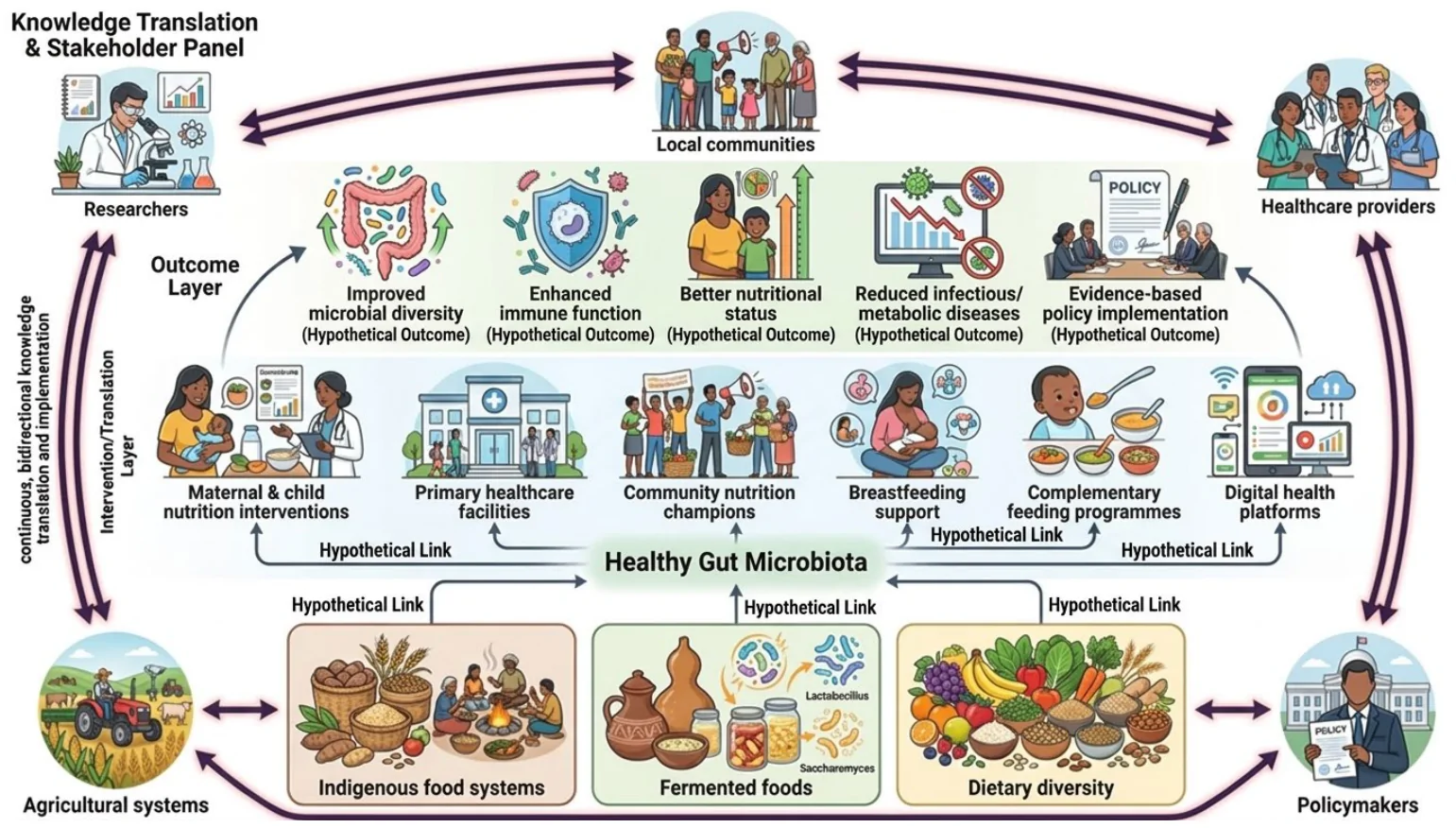
Microbiome-Informed Community Healthcare Framework (Created with FigureLabs). Conceptual framework illustrating the potential translation of microbiome science into community and primary healthcare. The framework depicts indigenous food systems, fermented foods, and dietary diversity as potential foundations for supporting a healthy gut microbiota, with maternal and child nutrition interventions delivered through primary healthcare facilities, community nutrition champions, breastfeeding support, complementary feeding programs, and digital health platforms. Hypothetical links are indicated throughout the figure, including potential relationships between dietary and community-based interventions, gut microbial diversity, immune function, nutritional status, and infectious and metabolic disease outcomes. The framework also illustrates potential pathways for evidence-based policy implementation and bidirectional interactions among researchers, healthcare providers, policymakers, agricultural systems, and local communities to support continuous knowledge generation, translation, implementation, and feedback. The depicted relationships and outcomes are conceptual and hypothesis-generating and should not be interpreted as established causal relationships or confirmed clinical effects.

**Figure 4:**
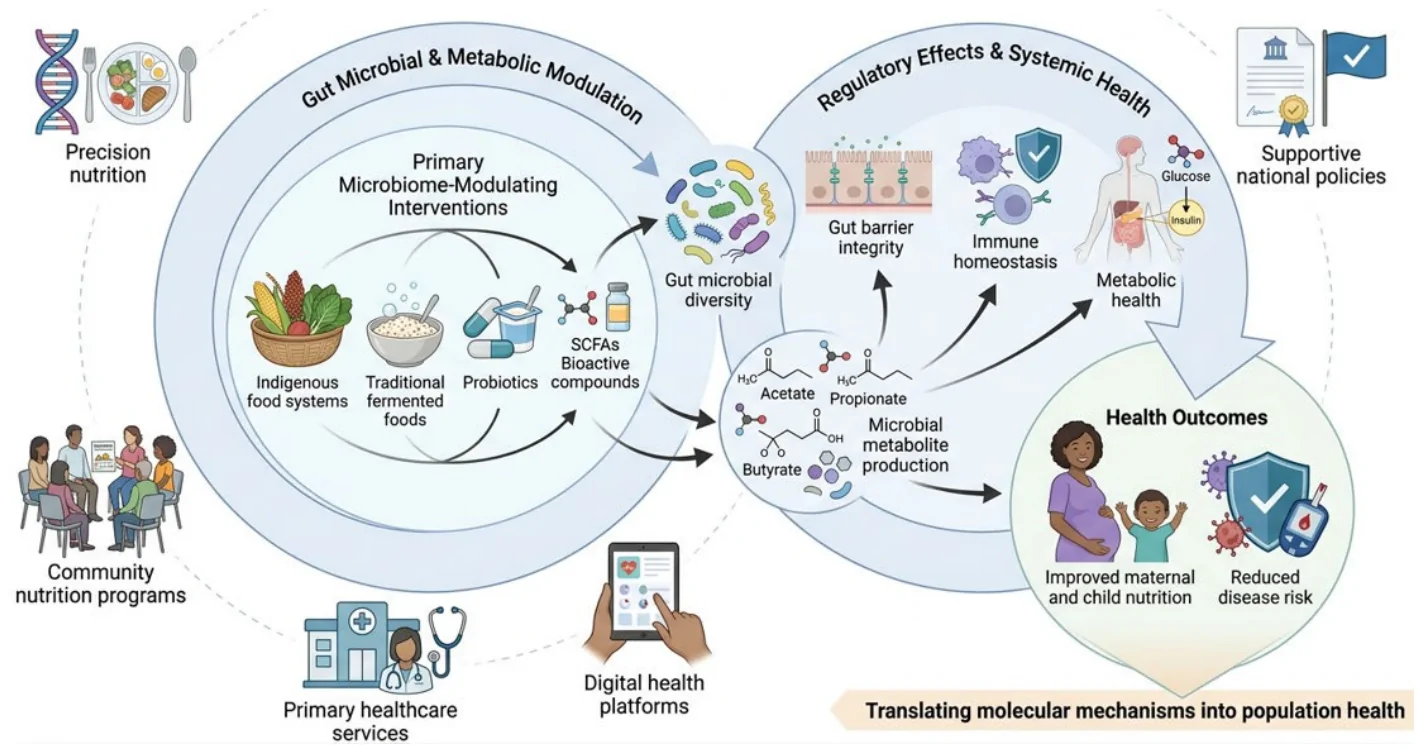
Proposed Microbiome-Informed Nutrition Framework (MINF): A conceptual and hypothesis-generating model. Conceptual framework illustrating the proposed Microbiome-Informed Nutrition Framework (MINF), showing indigenous food systems, traditional fermented foods, probiotics, and postbiotics as key microbiome-modulating interventions that may influence gut microbial diversity and microbial metabolite production, including SCFAs and other bioactive compounds. These microbial functions are linked to processes involving gut barrier integrity, immune homeostasis, and metabolic health, although the clinical effects of specific interventions require further validation. The framework distinguishes evidence-supported biological and nutritional components from proposed translational and implementation applications. The evidence-supported core links dietary exposures and substrates with microbial composition and function, microbial metabolites, and host–microbiome interactions. The framework also includes enabling systems such as precision nutrition, community nutrition programs, primary healthcare services, digital health platforms, and supportive national policies. These are presented as proposed translational applications rather than validated components of the framework. The model is intended to generate testable hypotheses and guide future research rather than imply established clinical effectiveness.

**Table 1:** Microbiome-associated conditions relevant to maternal, infant and child health: evidence and mechanisms.

Disease/Condition	Representative Microbial Alterations	Major Mechanisms/Associations	Key Health Consequences	Evidence Level/Study Design	Reference
Maternal diet, gut microbiota and pregnancy-related metabolic health	Dietary patterns were associated with differences in maternal gut microbiota. High-fiber and vegetarian diets were associated with increased *Roseburia* sp. and *Bifidobacterium* sp., whereas high-fat/Westernized diets were frequently associated with reduced microbial diversity and increased pro-inflammatory taxa, including *Collinsella* and members of *Lachnospiraceae*.	Associations between dietary composition, maternal microbiota and metabolic markers; maternal health status modified some diet–microbiota associations.	Potential relevance to gestational diabetes mellitus (GDM), obesity and hypertensive disorders of pregnancy.	Systematic review and correlational meta-analysis; 29 studies, 3077 pregnant women.	[47]
Childhood stunting	Differences in gut microbial composition between stunted and non-stunted children; higher abundance of *Bacteroidetes*/*Bacteroidota*, *Cyanobacteria*, *Lachnoclostridium*, *Faecalibacterium* and *Veillonella* in the stunted group in the studied populations.	Differences in microbial composition and SCFA production may be related to energy extraction and intestinal barrier function. However, the study notes that causality remains uncertain and that diet, sanitation and other environmental factors may contribute.	Growth faltering and impaired linear growth.	Human observational comparative study; 200 children (100 stunted, 100 non-stunted), 16S ribosomal ribonucleic acid (16S rRNA) sequencing and fecal SCFA analysis.	[48]
Obesity and metabolic dysfunction	Reduced microbiome richness and altered microbial ecology/functional potential were associated with a metabolome-informed obesity phenotype.	Bidirectional associations involving the microbiome, metabolites, lipids, amino acids, diet-derived metabolites and adipose-related metabolic dysfunction.	Insulin resistance, hyperinsulinemia, inflammation, fatty liver disease and other cardiometabolic abnormalities.	Human multi-omics observational cohorts with external validation; 1408 individuals in the primary cohort, 466 in an external cohort; additional bariatric surgery cohort (*n* = 75).	[49]
Allergic diseases	Alterations in early-life microbial colonization and microbial diversity have been associated with allergic disease development.	Microbiota–immune interactions may influence adaptive immune development, T-regulatory responses and immune tolerance.	Asthma, eczema and food allergy.	Narrative/review and mechanistic evidence; primary human and experimental studies discussed.	[50]
Diabetes mellitus, including gestational diabetes	Alterations in gut microbial composition and microbial metabolites have been linked with type 1 diabetes (T1D), type 2 diabetes (T2D) and GDM.	Proposed relationships involve gut-barrier impairment, inflammation, microbial metabolites and diet–host–microbiota interactions.	Impaired glucose regulation and metabolic dysfunction.	Narrative review synthesizing human, mechanistic and intervention evidence; direct maternal-child evidence should not be inferred from the review alone.	[51]
Inflammatory bowel disease (IBD)	Reduced abundance of *Faecalibacterium prausnitzii* in IBD compared with healthy controls.	Microbial dysbiosis may be associated with intestinal inflammatory processes and altered host–microbiome interactions.	Chronic intestinal inflammation, particularly Crohn’s disease and ulcerative colitis.	Systematic review and meta-analysis; 11 studies, 1180 participants.	[52]
Necrotizing enterocolitis (NEC) in preterm infants	Gut dysbiosis and altered microbial colonization have been implicated in NEC, although microbial patterns are influenced by prematurity, feeding and clinical factors.	Interaction between intestinal immaturity, dysbiosis, impaired mucosal immunity and nutritional practices.	Intestinal injury, morbidity and mortality among preterm/low-birthweight infants.	Narrative review of neonatal evidence; clinical, mechanistic and microbiome-related evidence synthesized.	[53]
Diarrheal disease	Enteric infections may disrupt gut microbial composition and colonization resistance, with expansion of potentially pathogenic/pro-inflammatory organisms.	Dysbiosis may be associated with impaired intestinal barrier function, altered immunity, inflammation and malabsorption.	Persistent diarrhoea and potential nutritional consequences in children.	Narrative review/chapter; human and mechanistic evidence synthesized.	[54]

**Table 2:** Key Molecular Mechanisms Underlying Microbiome–Host Interactions.

Mechanistic Component	Major Molecules/Pathways	Biological Function	Potential Health Relevance	References
Probiotics	*Lactiplantibacillus*, *Lacticaseibacillus*, *Bifidobacterium* and other beneficial microorganisms; bacteriocins and microbial metabolites	Modulation of intestinal microbial communities, competitive exclusion of pathogens, production of antimicrobial compounds and microbial metabolites, and interaction with host immune and epithelial systems	Potential contribution to gut microbial homeostasis and gastrointestinal, immune and metabolic health; effects are strain-, dose-, and context-specific	[68]
Postbiotics	Non-viable microbial cells and cell components, microbial metabolites, organic acids, bacteriocins and other bioactive compounds	Direct and indirect modulation of microbial communities; interaction with host pattern-recognition receptors; antimicrobial, barrier-protective and immunomodulatory effects	Potentially stable microbiome-targeted approaches for supporting intestinal barrier function and immune regulation; maternal and child applications require further clinical evaluation	[69]
Short-chain fatty acids (SCFAs)	Acetate, propionate and butyrate; free fatty acid receptor 3/G protein-coupled receptor 41 (FFAR/GPR41), G protein-coupled receptor 43 (GPR43) and G protein-coupled receptor 109A (GPR109A) signalling; histone deacetylase (HDAC) inhibition	Microbial fermentation products act as signalling molecules that regulate epithelial function, immune responses, energy metabolism and gut–host communication	Potential relevance to intestinal barrier function, immune homeostasis, glucose and lipid metabolism, and gut–host signalling	[70,71,72]
Succinate signalling	Microbial succinate and succinate-responsive host signalling	Functions as a microbial and host-derived metabolite and signalling molecule involved in microbiota–host and gut–immune tissue communication; influences intestinal epithelial cells, macrophages and dendritic cells	Potential role in intestinal homeostasis and immune regulation; its therapeutic relevance remains under investigation	[73]
Butyrate-producing microbiota	Butyryl-CoA pathway; *Faecalibacterium*, *Roseburia*, *Eubacterium*, *Anaerostipes*, *Coprococcus*, *Subdoligranulum* and *Anaerobutyricum*	Conversion of dietary substrates into butyrate; butyrate supports colonocyte energy metabolism, epithelial integrity and immune regulation	Potential contribution to gut barrier integrity, regulation of excessive inflammation and intestinal homeostasis	[74]
Gut barrier integrity	Tight-junction proteins including occludin, claudins and ZO-1; mucins; epithelial signalling pathways	Maintains intestinal epithelial integrity and regulates permeability between the gut lumen and systemic circulation	Reduced intestinal permeability and limitation of microbial-product translocation; potentially relevant to inflammatory and metabolic disorders	[75]
Immune regulation	Regulatory T cells (Tregs), regulatory B cells (Bregs), regulatory cluster of differentiation 8-positive T cells (CD8^+^ T cells), regulatory dendritic cells and other regulatory immune-cell populations	Regulation of immune activation and maintenance of immune tolerance and homeostasis	Potential contribution to control of excessive inflammation and immune dysregulation	[76]
Toll-like receptor (TLR) signalling	Toll-like receptor 2 (TLR2), toll-like receptor 4 (TLR4), toll-like receptor 1/Toll-like receptor 6 (TLR1/TLR6); pathogen-associated and damage-associated molecular patterns (PAMPs/DAMPs); regulatory molecules including suppressor of cytokine signaling 1 (SOCS-1) and tumor necrosis factor alpha-induced protein 3 (A20)	Recognition of microbial and endogenous molecular signals and initiation of innate immune signalling; regulation of physiological and pathological inflammatory responses	Host–microbe communication and immune regulation, with potential relevance to pregnancy-related processes	[77]
AMPK signalling	AMP-activated protein kinase (AMPK) and upstream/downstream metabolic signalling networks	Cellular energy sensing and restoration of energy balance during metabolic stress; regulation of metabolic processes	Potential relevance to glucose and lipid metabolism, obesity, diabetes and metabolic dysfunction	[78]
mTOR signalling	Mechanistic target of rapamycin (mTOR) signalling network	Integrates nutrient and growth-factor signals to regulate cell growth, protein synthesis, autophagy and metabolism	Relevant to growth, nutrient sensing and metabolic programming; dysregulation is implicated in metabolic and other chronic diseases	[79]
Mitogen-activated protein kinase (MAPK) signalling	Extracellular signal-regulated kinase (ERK), c-Jun N-terminal kinase (JNK) and p38 MAPK pathways	Regulation of cellular stress responses, inflammatory signalling, immune-cell functions and tissue responses	Potential role in modulation of inflammation, immune responses and tissue repair	[80]
Nuclear factor kappa-light-chain-enhancer of activated B cells (NF-κB) signalling	NF-κB transcription-control proteins and associated inflammatory signalling pathways	Regulation of inflammatory gene expression, immune responses, cell survival and proliferation	Central to regulation of inflammatory responses and therefore relevant to microbiome–host immune interactions	[81]

**Table 3:** Indigenous African foods and their potential microbiome-modulating relevance to maternal and child nutrition.

Indigenous Food/Food Group	Major Bioactive Components/Nutritional Features	Microbial or Microbiome-Relevant Effects	Population/Study Material	Study Design/Evidence Type	Microbiome Endpoint	Clinical/Nutritional Endpoint	Directness to Maternal–Child Health	Evidence Interpretation	Reference
Finger millet (*Eleusine coracana*)	Dietary fiber, polyphenols, flavonoids, phytic acid and other bioactive compounds	Potential prebiotic substrates; review discusses relationships involving *Faecalibacterium*, *Eubacterium*, *Roseburia*, *Bifidobacterium*, *Lactobacillus* and *Akkermansia muciniphila*	Experimental/review evidence; not specific to mothers or children	Narrative review integrating experimental and microbiome-related evidence	Microbiome-related evidence discussed; specific intervention endpoint not established in maternal/child populations	Potential relevance to glucose regulation, obesity, inflammation and metabolic health	Indirect	Mechanistic/review-based; hypothesis-generating for maternal–child application	[94]
Acha/Fonio (*Digitaria exilis*, *D. iburua*)	Dietary fiber, resistant/prebiotic carbohydrate fractions, amino acids, minerals and phytochemicals	Increased fiber and resistant starch may provide substrates for microbial fermentation; gut microbiota not directly assessed	Food samples/two fonio varieties	Experimental food-processing study	Not assessed	Nutritional, physicochemical, antioxidant and digestibility parameters	Indirect	Nutritional evidence; microbiome relevance remains hypothesis-generating	[95]
Bambara groundnut/fermented dawadawa	Protein, amino acids, minerals and phenolic compounds	Fermentation alters nutritional and antinutrient composition; gut microbiome not directly assessed	Food samples/fermented food products	Laboratory food-composition and fermentation study	Not assessed	Protein, amino acids, minerals and antinutrient composition	Indirect	Nutritional/food-processing evidence; microbiome benefits not demonstrated	[96]
Cowpea (*Vigna unguiculata*)	Protein, carbohydrates, raffinose, verbascose and other galacto-oligosaccharides	Processing and fermentation altered fermentability	18 healthy adults; cowpea porridge	Combined in vitro and in vivo study	Gut microbiome not directly assessed	Fermentability/flatulence index and gastrointestinal tolerance-related outcomes	Indirect—adult evidence	Human adult evidence; does not establish maternal–child microbiome benefit	[97]
African leafy vegetables, e.g., *Corchorus olitorius*	Phenolic compounds and antioxidant phytochemicals	Microbiome modulation not directly measured; biological activity observed in cultured colon-cell models	Plant extracts/cultured colon cells	Phytochemical and in vitro cell-model study	Not assessed	Cellular biological activity; no maternal/child clinical endpoint	Mechanistic/indirect	Preclinical evidence; not evidence of maternal or child health benefit	[98]
Fermented maize (Ogi) and other Nigerian fermented foods	Lactic acid bacteria (LAB) and fermentation-associated metabolites	LAB isolated from Ogi and Fufu included *Lactobacillus amylolyticus*, *L. plantarum* and *L. sakei.* Isolates demonstrated antimicrobial activity and characteristics relevant to starter/protective cultures, including tolerance to low pH and bile salts and bacteriocin/exopolysaccharides (EPS)-related properties	Nigerian traditional fermented foods, including Ogi and Fufu	Food microbiology study involving isolation, 16S rRNA identification and in vitro antimicrobial characterization of LAB	Food-associated microbial composition and LAB characteristics assessed; human gut microbiome not assessed	Food fermentation quality, antimicrobial activity and potential food-preservation functions; maternal–child clinical outcomes not assessed	Indirect	Food microbiology evidence; supports potential functional/food-safety relevance but is hypothesis-generating for maternal–child microbiome applications	[99]
Kunu (fermented cereal beverage)	Fermentation-associated LAB and antimicrobial metabolites	LAB isolated from Kunu, including *Lactobacillus plantarum*, *L. delbrueckii*, *L. fermentum*, *Pediococcus acidilactici* and *Leuconostoc mesenteroides*, showed inhibitory activity against toxigenic Aspergillus flavus	Kunu and LAB isolates; animal model for selected LAB activity	Food microbiology study with in vitro antifungal assays and an animal/preclinical model	Food-associated LAB and antifungal activity assessed; human gut microbiome not assessed	Potential food biopreservation and reduction of fungal contamination; no demonstrated maternal–child clinical benefit from Kunu consumption	Indirect	Food microbiology and preclinical evidence; hypothesis-generating rather than direct clinical evidence	[100]
Traditional fermented foods/fermented food systems	Fermentation-derived organic acids, microbial metabolites, nutrients and bioactive compounds	Fermentation microorganisms may function as “microfactories” that generate or transform nutrients and bioactive compounds; effects depend on microorganisms, raw materials and fermentation conditions	Fermented foods and fermentation systems; evidence drawn from diverse food systems	Narrative review of fermentation-enabled wellness foods and microbial/enzyme-mediated processing	Microbial fermentation processes and metabolites discussed; no specific human gut microbiome endpoint established	Potential nutritional and functional-food relevance; clinical benefits require product-, strain- and population-specific validation	Indirect	Review-level food and mechanistic evidence; supports biological plausibility but does not establish maternal–child clinical effects	[101]
Dietary patterns and indigenous food consumption in African and other populations	Dietary diversity and food-specific nutrients, dietary fibres and bioactive components	Dietary factors, including vegetables, red meat, dairy and overall diet quality, mediated differences in several gut bacterial genera, including *Lachnospira, Ruminococcus-1* and *Bifidobacterium*	5267 healthy adults from a multiethnic population	Large human observational cohort with 16S rRNA sequencing and dietary mediation analyses	Gut microbiome composition and bacterial taxa; diet-associated differences in microbial composition were assessed	Dietary intake and overall diet quality; no maternal, pregnancy or child clinical endpoint	Indirect—adult population	Human adult microbiome evidence; demonstrates diet–microbiome associations but is not specific to African indigenous foods, pregnancy or childhood	[102]
Fermented foods	Fermentation-derived microorganisms, organic acids, microbial metabolites and bioactive compounds	A high-fermented-food diet increased gut microbiome diversity and decreased inflammatory markers; a high-fibre diet increased microbiome-encoded carbohydrate-active enzymes without a corresponding increase in overall diversity	Healthy adults participating in a controlled dietary intervention	Randomized prospective human dietary intervention; n = 18 per intervention arm, 17-week intervention	Gut microbiome diversity and microbiome-encoded carbohydrate-active enzymes	Inflammatory markers/immune status	Indirect—adult population	Direct human intervention evidence for fermented-food effects on the gut microbiome and inflammatory state, but not specific to African fermented foods, pregnancy or childhood	[103]
Indigenous food systems of tribal communities	Diverse indigenous foods, dietary patterns and food-system components; dietary diversity and indigenous food consumption	Proposed assessment of gut microbiota profiles in women alongside dietary patterns, indigenous food consumption and nutritional outcomes; the study does not establish that a particular food causes a specific microbiome change	Women and indigenous tribal communities in three Indian tribal communities	Prospective observational mixed-methods study protocol, with planned three-year longitudinal follow-up	Planned gut microbiota profiling; no completed microbiome outcome reported in the protocol	Planned dietary, nutritional and food-system outcomes; no completed clinical outcome reported	Indirect	Study-protocol evidence; potentially informative for indigenous food–microbiome research but hypothesis-generating and not evidence of a demonstrated maternal–child effect	[104]

**Table 4:** Traditional African fermented foods, associated microorganisms and potential microbiome-relevant functions.

Fermented Food	Primary Raw Material	Predominant/Relevant Microorganisms	Major Bioactive Compounds/Fermentation-Related Attributes	Population/Study Material	Study Design/Evidence Type	Microbiome Endpoint	Clinical/Nutritional Endpoint	Directness to Maternal–Child Health	Evidence Interpretation	Reference
Ogi	Maize; traditionally also sorghum or millet	LAB, including *Lactobacillus*/*Lactiplantibacillus* sp.; yeasts	Lactic acid and other fermentation metabolites; LAB-associated antimicrobial/functional properties	Fermented food/microbial isolates	Food microbiology; isolation and characterization; in vitro testing	Human gut microbiome not assessed	Acid/bile tolerance and cholesterol-related activity in vitro; no maternal/child endpoint	Indirect/mechanistic	Food microbiology and in vitro evidence; microbiome and clinical effects unestablished	[105]
Fura	Millet	LAB including *L. fermentum*, *Weissella confusa*, *Pediococcus acidilactici*, *L. reuteri*, *L. salivarius* and *L. paraplantarum*	Lactic acid and fermentation-associated microbial metabolites	Fura from eight production sites in northern Ghana; 862 LAB isolates	Food microbiological characterization; phenotypic/genotypic identification	Microbial composition/succession within the fermented food; human gut microbiome not assessed	Nutritional/clinical outcomes not assessed	Indirect/not directly applicable	Food microbiology evidence; potential microbiome relevance is hypothesis-generating	[106]
Dawadawa (Iru)	African locust bean (*Parkia biglobosa*)	*Bacillus*, *Staphylococcus*, *Streptococcus*, *Lactobacillus*; *B. subtilis* predominant	Protein, fiber, minerals and fermentation-associated metabolites	Food samples from six communities in northern Ghana	Multi-site food characterization; proximate/mineral analysis and 16S rRNA sequencing	Bacterial diversity/composition of the fermented food	Protein/mineral bioavailability-related food characteristics; no human clinical endpoint	Indirect	Food microbiology evidence; direct human gut-microbiome effects not demonstrated	[107]
Traditional African fermented foods	Cereal, dairy and plant-based substrates	Mainly LAB and yeasts, with other microorganisms depending on product	Organic acids, microbial metabolites, vitamins, peptides and other bioactive compounds	Fermented food systems across reviewed studies	Narrative/review synthesis	Product/strain-dependent microbiome relevance; no single maternal–child endpoint	Potential nutritional/functional-food relevance; clinical benefits require validation	Indirect	Review-level evidence; product- and strain-specific; hypothesis-generating	[108]
Selected Nigerian indigenous fermented foods (ogi, pap, kunu, palm wine, zobo)	Various cereal/plant substrates	LAB including *L. fermentum*, *Lactococcus lactis*, *Weissella confuse*, *Enteroccoccus gallinarum*, and other isolates	Strain-dependent antimicrobial/antifungal activities and fermentation-related properties	Food isolates; *Drosophila melanogaster* model	16S rRNA identification, in vitro probiotic/antimicrobial testing and in vivo animal-model validation	Human gut microbiome not assessed	Host-protection findings in Drosophila; no maternal/child clinical endpoint	Mechanistic/preclinical	Preclinical evidence; does not establish maternal–child clinical benefit	[109]

**Table 5:** Representative microbiome and maternal–Child nutrition studies from Africa.

Country	Population/Sample	Sample Size	Study Design/Evidence Type	Research Focus	Microbiome Endpoint	Clinical/Nutritional Endpoint	Key Findings	Directness to Maternal–Child Health	Evidence Interpretation	Reference
Nigeria	Traditional fermented foods; no defined human participant population	Not applicable	Narrative review/food microbiology evidence	Indigenous fermented foods, LAB, food microbiology and nutrition	Not assessed in human participants; microbial diversity and LAB characteristics of fermented foods were reviewed	Nutritional and biotechnological potential of fermented foods; clinical outcomes not assessed	Traditional Nigerian fermented foods, including ogi, gari, fufu, lafun, kunu-zaki and masa, harbour diverse LAB with potential nutritional and biotechnological value, including B-group vitamin production	Indirect	Food-system/microbiological evidence; hypothesis-generating for maternal–child nutrition	[125]
Kenya	Infants in a rural birth cohort	n = 100 infants	Prospective birth cohort/observational study	EED, breastfeeding, seasonality, infant growth and intestinal bacterial overgrowth	Small intestinal bacterial overgrowth; gut microbiota diversity was examined in relation to EED and growth	EED biomarkers, stunting/linear growth; 33% of assessed infants were stunted at 9 months	Cessation of exclusive breastfeeding and seasonality were associated with EED, whereas small intestinal bacterial overgrowth was not strongly associated; microbiota diversity was not strongly associated with EED or linear growth impairment	Direct for child health; indirect for microbiome-informed nutrition	Direct human child evidence with microbiome-related assessment; supports breastfeeding and early-life nutrition research but does not establish a microbiome-mediated causal effect	[126]
Ethiopia	Children under 2 years and their mothers	n = 1054 mother–child pairs	Observational/cross-sectional dietary study	Infant feeding practices, complementary feeding and nutritional status	Not assessed	Complementary-feeding practices; stunting, wasting and underweight	84.7% of children were introduced to complementary foods at six months; cereals, vegetables and fruits were commonly introduced, while indicators of undernutrition were also observed	Direct for maternal–child nutrition; indirect for microbiome	Human maternal–child nutritional evidence; microbiome implications remain proposed rather than demonstrated	[127]
Ghana	Children aged 6–23 months	n = 384 children	Observational/cross-sectional study	Commercial infant cereals, complementary feeding and micronutrient intake	Not assessed	Micronutrient intake and complementary-feeding practices	Commercial infant cereals contributed substantially to recommended intakes of several micronutrients, but reliance on these products may reduce emphasis on locally available nutrient-dense foods	Direct for child nutrition; indirect for microbiome	Human child nutritional evidence; supports dietary-diversity considerations but does not demonstrate microbiome effects	[128]
South Africa	Rural and urban African participants from Soweto and Bushbuckridge	n = 167 participants	Observational/cross-sectional comparative cohort	Rural–urban transition, diet/lifestyle, gut microbiome and obesity	Gut microbiome composition, community clustering and taxa including *Prevotella*	Obesity status and lifestyle/geographic transition	Gut microbiome composition differed between urban and transitioning rural populations; geographic location was more important for microbiome clustering than lean/obese status, with some taxa associated with obesity	Indirect	Human adult microbiome evidence; relevant to African diet–microbiome transitions but not maternal–child-specific	[129]
Kenya	Traditional fermented foods; no defined human participant population	Not applicable	Narrative review/food microbiology evidence	Traditional fermented foods, microbial composition and food-system nutrition	Microbial communities in fermented foods; consumer gut microbiome not assessed	Food safety, nutritional quality and product consistency; human clinical outcomes not assessed	Traditional Kenyan fermented foods, including cereal-based uji and fermented milk products such as mursik and suusac, harbour diverse microbial communities; locally appropriate starter cultures may improve safety and consistency while retaining traditional characteristics	Indirect	Food microbiology/food-system evidence; translational and hypothesis-generating rather than direct maternal–child evidence	[130]

**Table 6:** Potential community-based applications of microbiome-informed maternal and child nutrition.

Application Area	Target Population/Setting	Evidence Base/Study Design	Microbiome Endpoint	Maternal–Child Clinical/Nutritional Endpoint	Proposed/Potential Application	Directness to Maternal–Child Health	Evidence Status	References
Nutrition education and indigenous foods	Indigenous populations, mothers, caregivers and communities	Scoping review of interventions/programmes using native foods	Not directly assessed across the evidence base	Health and nutrition outcomes reported across diverse programmes; outcomes vary by intervention	Integration of indigenous foods, dietary diversity and culturally appropriate food practices into nutrition education	Indirect	Emerging/evidence-informed application; microbiome effects require evaluation	[135]
Community nutrition champions	Pregnant/lactating women, mothers, caregivers and communities in low- and middle-income countries (LMICs)	Systematic review and meta-analysis of community home-visit and peer-group nutrition interventions	Not assessed as a consistent endpoint	Breastfeeding, complementary feeding, dietary diversity and child nutritional outcomes	Use of community health workers, peer educators and women’s groups to deliver nutrition support and education	Direct for maternal–child nutrition; indirect for microbiome	Evidence-supported for nutrition delivery; microbiome application is proposed	[136]
Primary healthcare integration	Mothers, infants and children using primary healthcare services	Quasi-experimental study protocol/health-service integration evidence	Not assessed	Maternal and child health service integration and postpartum family-planning outcomes	Integration of nutrition and maternal–child health services as a potential platform for microbiome-informed nutrition counselling	Direct for maternal–child health systems; indirect for microbiome	Implementation strategy; microbiome-informed component is proposed	[137]
Digital health and mobile nutrition support	Mothers, caregivers and maternal–child health service users	Cross-regional mHealth evidence/programme-based evidence	Not assessed	Maternal–child health information, monitoring, counselling and service engagement	Use of mobile platforms, SMS and digital tools to support nutrition education, monitoring and maternal–child health engagement	Direct for maternal–child health delivery; indirect for microbiome	Emerging/proposed application	[138]
Personalized nutrition	Individuals requiring tailored dietary guidance; potentially mothers and children in future applications	Narrative review/emerging personalized-nutrition evidence	Microbiome and metabolic information proposed as components of personalization; maternal–child microbiome endpoints not established	Dietary and metabolic outcomes, particularly in chronic disease contexts	Future integration of nutritional, microbiome and metabolic information into individualized maternal–child dietary recommendations	Indirect	Emerging/hypothesis-generating for maternal–child application	[139]
Digital dietary assessment	Individuals and populations requiring dietary monitoring; potentially maternal–child settings	Review of digital technologies for dietary assessment	Not directly assessed	Dietary intake/food-consumption assessment rather than direct maternal–child clinical outcomes	Smartphone, image-based and biomarker-supported dietary assessment to improve characterization of maternal and child dietary exposures	Indirect	Emerging methodological application	[140]
Indigenous food and microbiome-informed nutrition	African populations, including potential maternal–child populations	Narrative/review evidence on African diets and gut microbiota	Gut microbiome–diet relationships discussed; maternal–child intervention endpoints not established	Human health and metabolic outcomes discussed across the literature; direct maternal–child outcomes not established	Further evaluation of traditional African diets rich in plant foods, dietary fibre and fermented foods as potential components of sustainable nutrition strategies	Indirect	Hypothesis-generating/proposed	[141,142]
Diet–microbiome-informed nutrition and policy translation	Communities, health systems and populations; potential maternal–child programmes	Narrative/methodological guidance on dietary-pattern–microbiome integration	Depends on studies incorporated into the framework; no direct intervention endpoint established	Potential integration into dietary guidelines and nutrition programmes; clinical effects require evaluation	Translation of diet–microbiome evidence into food-based dietary guidelines, maternal–child nutrition programmes and community nutrition policies	Indirect	Proposed translational framework	[143]

**Table 7:** Emerging technologies in microbiome-informed maternal and child nutrition.

Technology/Innovation	Primary Application	Potential Contribution to Maternal and Child Nutrition	Current Challenges	Reference
Artificial Intelligence (AI)	Machine learning and predictive modelling	Disease-risk prediction, microbial biomarker identification, and research into personalized nutrition	Limited African datasets, algorithm validation, data quality, bias and interpretability	[151]
Metagenomics	Identification of microbial taxa and functional genes	Characterization of maternal–infant microbiome composition and microbial functions	High sequencing cost and bioinformatics expertise	[152]
Metabolomics	Analysis of microbial metabolites such as SCFAs and bile acids	Identification of functional metabolic pathways and nutritional biomarkers	Standardization of analytical platforms	[153]
Proteomics	Protein expression profiling	Identification of host–microbe functional interactions	Complex data interpretation	[154]
Transcriptomics	Gene expression analysis	Understanding host and microbial responses to dietary interventions	Temporal variability of gene expression	[155]
Precision microbiome nutrition	Microbiome-guided dietary interventions	Potentially individualized maternal and child nutrition strategies	Limited clinical validation and implementation evidence	[156]
Microbiome engineering	Targeted manipulation of microbial communities	Development of next-generation probiotics and microbial therapeutics	Regulatory, safety, and ethical considerations	[157]
Personalized nutrition	Integration of microbiome, diet, genetics, and clinical data	Potentially more targeted dietary recommendations	Accessibility, affordability, and health equity	[158]

**Table 8:** Major organizations and their roles in microbiome-informed nutrition.

Organization	Primary Role	Relevance to Microbiome-Informed Nutrition	Reference
World Health Organization (WHO)	Global public health guidance	Promotes maternal and child nutrition, breastfeeding, food safety, and evidence-based nutrition policies	[162]
Food and Agriculture Organization (FAO)	Food systems and nutrition	Supports sustainable food systems, dietary diversity, food security, and guidance on probiotics and fermented foods	[163]
United States Food and Drug Administration (FDA)	Regulation of foods, biologics, and therapeutics	Provides regulatory oversight relevant to the safety, manufacturing, and regulatory classification of applicable microbiome-related products	[164]
European Food Safety Authority (EFSA)	Scientific risk assessment	Evaluates food safety, novel foods, and health claims within the European Union	[165]
National Ministries of Health	National policy implementation	Potential integration of validated microbiome-related evidence into maternal and child health programs and dietary guidelines	[166]
National Food Regulatory Agencies	Product regulation	Ensure applicable quality, safety, labeling, and monitoring requirements for probiotic, postbiotic, and functional food products	[167]

**Table 9:** Emerging innovations and their potential applications.

Innovation	Primary Function	Potential Application in Maternal and Child Nutrition	Reference
Artificial intelligence	Predictive analytics and machine learning	Microbiome classification, nutritional risk assessment, and potentially personalized dietary support	[178]
Multi-omics integration	Functional characterization of host–microbe interactions	Biomarker discovery and mechanistic understanding of diet–microbiome–host interactions	[179]
Postbiotic therapeutics	Delivery of microbial-derived bioactive compounds	Potentially stable alternatives to selected live probiotic preparations; effects remain preparation- and context-specific	[180]
Precision nutrition	Individualized dietary interventions	Potentially personalized maternal and infant nutrition strategies; clinical validation remains limited	[181]
Personalized microbial therapies	Targeted microbiome modulation	Potential applications in microbiome-associated disorders; maternal and child health effectiveness remains to be established	[182]
Digital health technologies	Remote monitoring and decision support	Nutrition counseling, dietary tracking, and community health support, including mobile health and telehealth applications	[183]

## Data Availability

No new data were created or analyzed in this study. Data sharing is not applicable to this article.
